# Influence of Clinical and Sociodemographic Variables on Health-Related Quality of Life in the Adult Population with Long COVID

**DOI:** 10.3390/jcm12134222

**Published:** 2023-06-22

**Authors:** Mª Pilar Rodríguez-Pérez, Patricia Sánchez-Herrera-Baeza, Pilar Rodríguez-Ledo, Elisabet Huertas-Hoyas, Gemma Fernández-Gómez, Rebeca Montes-Montes, Marta Pérez-de-Heredia-Torres

**Affiliations:** 1 Group in Evaluation and Assessment of Capacity, Functionality and Disability of Universidad Rey Juan Carlos (TO+IDI), Department of Physical Therapy, Occupational Therapy, Physical Medicine and Rehabilitation, Research, Universidad Rey Juan Carlos, 28922 Alcorcón, Spain; pilar.rodriguez@urjc.es (M.P.R.-P.); elisabet.huertas@urjc.es (E.H.-H.); gemma.fernandez@urjc.es (G.F.-G.); rebeca.montes@urjc.es (R.M.-M.); marta.perezdeheredia@urjc.es (M.P.-d.-H.-T.); 2 COVID Persistent Working Group of the Sociedad Española de Medicos de Familia (SEMG), Department of General Medicine, Lugo, a Mariña and Monforte de Lemos Health Area, 27002 Lugo, Spain; prodriguezl@semg.es

**Keywords:** long COVID, quality of life, persistent symptoms, long haulers

## Abstract

Worldwide, about 10 percent of patients affected by long COVID require appropriate follow-up and intervention. The main objective of this study was to analyze the long-term impact of mild long COVID in the adult population, and to determine the effect of clinical and sociodemographic variables on health-related quality of life in those affected. Methods: A cross-sectional descriptive study of a sample of Spanish adult patients with persistent COVID-19 symptoms at least three months after diagnosis. Data collection took place between April and July 2021. The health-related quality of life of the sample was low, with worse results in the physical component summary (PCS) 24.66 (SD = 4.45) compared to the mental component summary (MCS) 45.95 (SD = 8.65). The multi-regression analysis showed significant differences by sex in the dimensions of physical functioning (*p* = 0.040); bodily pain (*p* = 0.036); and health transition (*p* = 0.018). Additionally, a longer time since infection had a significant effect on physical functioning (*p* = 0.039); general health (*p* = 0.037); vitality (*p* = 0.034); and general health transition (*p* = 0.002). The effect of occupational imbalance was significant for all dimensions. Conclusions: people with long COVID have a reduced quality of life. Sex, time since infection, and occupational imbalance are predictors of a worse quality of life.

## 1. Introduction

Severe acute respiratory syndrome coronavirus 2 (SARS-CoV-2) is the causative agent that results in acute COVID-19 infection. Research has shown that mild COVID-19 disease is present in up to 80% of cases [1,2]. In addition, long-term complications can occur after the acute phase of COVID-19 disease following recovery from the acute effects of the infection [3]. Some people continue to experience symptoms beyond the initial acute phase of the disease with long term effects from their infection, known as long COVID [2,4]. Thus, Greenhalgh et al. 2020 [5] defined long COVID as the persistence of symptoms beyond 12 weeks of symptom onset. Between 11% and 24% of patients with COVID-19 may experience long-term symptoms even three months after the onset of COVID-19 disease. The hypothesized etiopathogenesis of COVID-19 is that it may be driven by long-term tissue damage or pathological inflammation (due to viral persistence, immune dysregulation, and autoimmunity) [6]. There is scarce evidence for patients who suffered a mild COVID-19 infection and were treated in an outpatient setting. This is potentially contradictory as, according to the available data, most cases of individuals with acute COVID-19 experience a mild disease [7], and therefore, further studies in this population are required. The predominant profile of individuals with long COVID, is that of a woman with a mean age of 43 years and no previous major health problems [8]. Conversely, people with long COVID who were hospitalized in the acute phase of COVID-19 had a higher prevalence of comorbidity and previous pathologies [9]. The impact of the persistence of symptoms beyond the clinical outcome means that the individual’s physical, mental, social, and emotional functioning is affected [10], which has a major health and economic impact [11]. Previous research on persistent COVID has focused on characterizing persistent symptoms and the pathophysiology of the disease as well as the need for further research because of the great impact on quality of life [4] and occupational performance [9,12], months after the diagnosis. Thus, it is important to analyze performance limitations, together with the degree of occupational balance, i.e., the ability for a person to distribute their activities, together with time-management and decision-making, as this is fundamental for the individual’s autonomy [13]. Occupational balance can be defined as “The individual’s subjective experience of having “the right mix” of occupations in his or her occupational pattern. This definition can be used from various perspectives: occupational areas, occupations with different characteristics, and time use” [14]. It has been previously shown that occupational imbalance directly causes high levels of stress and impacts the individual’s health, however a balanced participation contributes to the maintenance of people’s health and well-being [15].

Due to the novelty of the disease, there is a lack of evidence regarding the evolution of those affected [16]. Thus, previous studies have monitored the number of symptoms [17], the lower health status of those with long COVID compared to that of the control population [18], and the risk factors for long COVID-19, such as being a female, vaccination status, and age [9]. However, the effect of long COVID on quality of life has not yet been sufficiently analyzed; therefore, it is necessary to research the limitations encountered in order to design an appropriate intervention and rehabilitation program to improve both quality of life and autonomy [13].

This study sought to identify the impact of long COVID on health-related quality of life to determine the effect of clinical and sociodemographic variables, and to explore the relationship with the participants’ occupational balance by performing a secondary analysis based on the previously published cross-sectional descriptive design by Rodriguez-Perez et al. [19].

## 2. Materials and Methods

### 2.1. Design

A descriptive cross-sectional study was conducted on a sample of Spanish adult patients presenting with persistent COVID-19 symptoms for three months or longer. The guidelines of the Strengthening the Reporting of Observational Studies in Epidemiology (STROBE) checklist were followed [20]. This study was approved by the Ethics Committee of University Rey Juan Carlos (170120210212) and is framed within the Spanish Research Network on Persistent COVID (REiCOP). Data collection, processing and transfer were completed in accordance with the provisions of the Declaration of Helsinki [21], and current Spanish regulations on personal data protection. Furthermore, prior to participation, each participant signed an informed consent form.

### 2.2. Sample

Data collection took place between April to July 2021. The survey method was adopted and applied through videoconference with participants. The sample criteria were determined in consensus with the Spanish Society of General and Family Physicians (SEMG), based on previous international studies [22]. The sample was selected using simple random sampling with all voluntary participants who met the criteria using the 2022 Quick-Calcs GraphPad software system (GraphPad Software, LLC, San Diego, CA, USA). The inclusion criteria consisted of people between 30 and 50 years of age, diagnosed with acute-phase COVID-19 disease via Polymerase Chain Reaction (PCR) and/or positive serology; who did not require hospitalization in the acute phase of illness; with persistent COVID-19 symptomatology determined by medical diagnosis and not attributed to another cause; for three months or longer; adequate ability to communicate for the purpose of collecting clinical data; and no previous pathologies; additionally, due to the health regulations in place at the time, the participants were non-vaccinated. The exclusion criteria consisted of receiving rehabilitation treatment for COVID-19 at the time of the assessment, not having the necessary technology to conduct the interview, and failure to accept and sign the informed consent form.

### 2.3. Procedure

**Figure 1:**
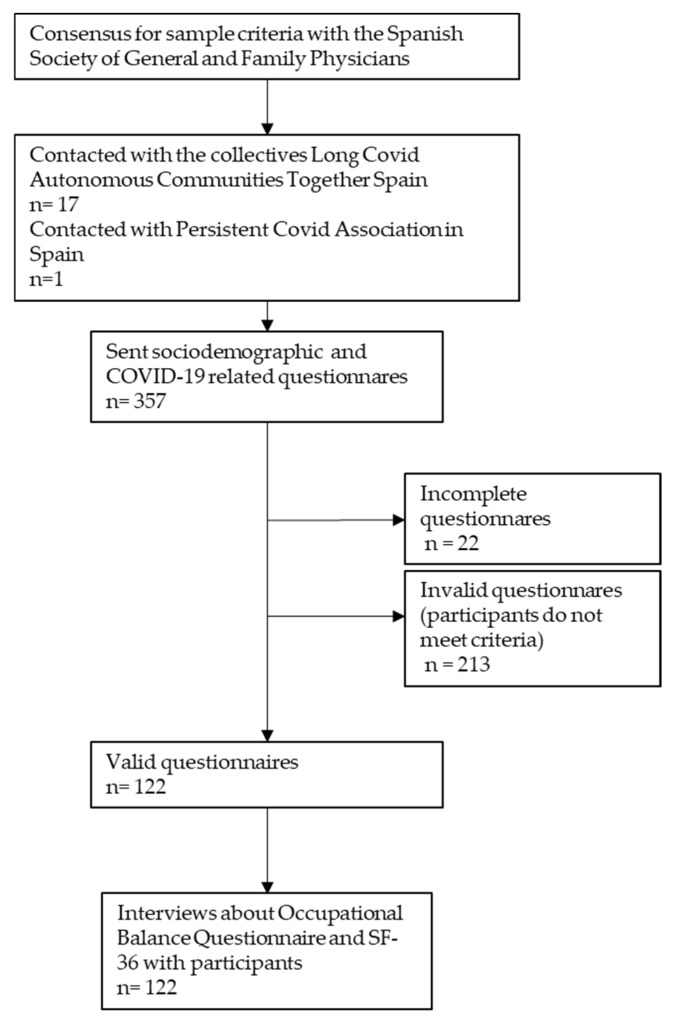
Data collection.

Before conducting the study, the researchers established agreements with the Spanish Society of General and Family Physicians (SEMG), the Persistent COVID Association in Spain (ACPE) and representatives of the collective “Long COVID Autonomous Communities Together Spain (ACTS)”. Thereafter, Long COVID ACTS conveyed the study information to the regional collectives of each community, who, in turn, disseminated the information to each of the affected patients who voluntarily showed their interest in participating in the study. The form gathered contact and sociodemographic data, COVID-19 diagnostic test data, time of evolution since diagnosis, symptomatology, employment status, and acceptance of the study. Once the participant had completed the form and accepted the informed consent, the investigator conducted a videoconference call. During the interview, both the Occupational Balance Questionnaire and SF-36 health questionnaire were administered. These scales were administered considering the participant’s situation at the time, and the same questions were also administered referring to their situation prior to the disease. Subsequently, the data were stored in a digital notebook that was solely available to the principal investigator. The study protocol is published elsewhere in detail [19] (Figure 1).

### 2.4. Measures

The Occupational Balance Questionnaire (OBQ) [23] measures occupational balance in relation to a person’s current situation and daily life. Thus, it assesses the ability to manage the amount and variability of tasks within an occupation while preserving personal preferences, as well as the ability to maintain a strong sense of self-identity through participation in meaningful occupations based on personal values. The OBQ particularly focuses a person’s satisfaction with the range and variability of occupations and provides a global picture of one’s own occupational balance [24]. It consists of 13 items that are scored using an ordinal response scale from 0 to 5 points according to the degree of agreement. The final score ranges from 0 to 65, where a higher score indicates a better occupational balance. A notable advantage of this questionnaire is that it does not focus on a single classification of activities, rather it presents different statements in a global way with several alternatives in reference to a wide range of activities that the individual may have. The main objective is to explore the balance between different types of occupations, the significance of the occupations for the person, the use of time, and how the patient feels about these occupations. Thus, a wide variety of occupations are represented, including physical, social, intellectual, leisure, and other activities [23]. This tool has demonstrated adequate psychometric properties, making it a reliable instrument for measuring occupational balance. Moreover, a Spanish version has been adapted and validated [24].

The impact on health-related quality of life (HRQoL) was measured using the 36-item Short-Form Health Survey (SF-36) [25]. The SF-36 was developed to measure relevant generic health concepts. It is a 36-item scale, measuring the following domains: physical functioning, physical role limitations, bodily pain, general health perceptions, social functioning, emotional role limitations, mental health, and the transformation in health status compared to the previous state. The raw scores are translated into transformed scores and each dimension is given a percentage from 0 to 100, the higher the percentage, the better the health status. The aggregation of the eight subdomain scores enables the calculation of two summary scores: the physical summary component (PCS) scores and the mental summary component (MCS) The PCS is calculated by positively weighting the four subscales in the physical domain (physical functioning, role physical, bodily pain and general health) and the remaining psychological domain subscales negatively [26]. In contrast, the MCS is calculated by positively weighting the four mental domain subscales (mental health, vitality, social functioning and role emotional), and negatively weighting the four physical domain subscales. Previous studies published to date have shown that scores above or below 50 indicate better or worse health status, respectively, than the mean of the reference population [27]. This questionnaire is psychometrically sound and has been validated and adapted to the Spanish population [28]. The SF-36 is designed to be self-administered. Recent studies have used this questionnaire with population affected by the COVID-19 pandemic [29].

### 2.5. Data Analysis

Concerning qualitative variables, the number of cases present in each category and the corresponding percentages were calculated. In addition, in terms of quantitative variables, the mean and standard deviation were calculated with transformed scores subscales. To calculate PCS and MSC values, scores for each of the eight domains were extracted and standardized using a z-score transformation. They were then multiplied by 10 and added to 50 to generate normalized scores for each domain and aggregated using factor score coefficients and creating normalized scores for each component summary [30]. Correlations between variables were studied using Pearson’s correlation coefficient with the raw scores. To determine the possible effect of demographic, clinical and scale variables, multivariable linear regression models were performed for the dimensions scores. The statistical analysis was performed with SPSS 27.0 for Windows (Copyright© 2013 IBM SPSS Corp., Armonk, NY, USA). Statistical significance was set at *p* < 0.05.

## 3. Results

The final study sample consisted of 122 patients from 35 Spanish territories, presenting with persistent multiple and multisystemic symptomatology. As shown in Table 1, up to 77.9% (*n* = 95) were women and 22.1 % (*n* = 27) were men, aged between 30 and 50 years with a mean age of 43.5 years (SD = 5.8). Regarding the time since infection, the mean time was 10.88 months (min.–max.:4–16, SD = 3.33).

Table 2 shows the means and standard deviations of the scores for the SF-36 subscales. The health-related quality of life of the sample was low, with worse results in the physical component summary (pcs; 24.66 [sd = 4.45]): physical functioning 27.50 (sd = 20.40); role of physical limitations 5.12( sd = 16.99); general health 29.51 (sd = 16.23); bodily pain 36.52(sd = 22.04) than in the mental component summary (mcs; 45.95 [sd = 8.65]): vitality 22.25 (sd = 20.71); mental health 59.30 (sd = 14.94); social functioning 39.45 (sd = 17.53); and role of emotional limitations 62.81(sd = 46.98). The health transition subscale scored an average of 7.17 (sd = 11.35).

Table 3 shows the means (standard deviations) and correlations between the scales (raw scores). Occupational balance measured using the OBQ correlated positively and significantly with all dimensions of the SF36, except with physical role limitations.

To determine the possible effect of demographic and clinical variables and the OBQ scale on the dimensions of the SF36 scale, multivariable linear regression models were calculated, considering physical functioning, role physical, general health and bodily pain as the dependent variables (Table 4). Regarding physical functioning, the variables with a significant effect were sex (*p* = 0.040), time of evolution (*p* = 0.039), and occupational balance (*p* < 0.001). The results revealed that for general health, time since infection (*p* = 0.037), and occupational balance (*p* < 0.001) were significant. For bodily pain, sex (*p* = 0.036) and OBQ (*p* = 0.003) were significant. None of the independent factors contributed to the role of physical health.

Regarding the dimensions related to mental health (Table 5), the results showed that time since infection had a statistically significant effect (*p* = 0.034). Additionally, for the four dimensions, the variable with a statistically significant effect was occupational balance, where a lower score was related to greater limitations for the following dimensions: vitality (*p* < 0.001) role of emotional limitations (*p* = 0.001), mental health (*p* = 0.005) and social functioning (*p* < 0.001).

In the dimension related to health transition (Table 6), the variables with a significant effect were sex (*p* = 0.018), time since infection (*p* = 0.002) and occupational balance (*p* < 0.001).

## 4. Discussion

This study shows the impact of mild persistent COVID on HRQoL. Our findings reveal low HRQOL scores compared to normative data of people in the same age range and similar characteristics [31]. The available evidence regarding the HRQoL of people with Long COVID remains scarce; however, affected individuals continue to present a wide range of symptoms [32], hampering the return to their previous normal life. In line with our results, Garrigues et al. [33] found an impact on quality of life in dimensions measured by the EQ-5D among previously hospitalized patients with persistent symptoms. Similarly. Arnold et al. [29] also administered the SF-36 to hospitalized patients with COVID-19 and showed a decline in HRQoL in all domains compared to age-matched population norms. Similar previous studies [32] have based their analyses on patients with acute COVID-19. However, to date, we have not found any studies focusing on individuals with persistent COVID who presented with mild SARS-CoV-2 infection.

The regression model showed that sex had a significant effect on the domains of physical functioning, bodily pain, and health transitions. Therefore, women had worse scores on the HRQOL compared to men. These scores may be in line with previous studies [34] where men obtained better scores on the HRQOL compared to women. However, these studies were only focused on hospitalized patients. Time since infection was another significant variable for vitality, and general health. Thus, a longer time of disease evolution was significantly correlated with lower HRQoL scores, and worse self-perceived general health. To the best of our knowledge, there is no data on the evolution of HRQOL in people with long-term persistent COVID. Thus, more recent studies have focused on accounting for long-term symptoms [4] and improvement of symptoms [6], and less on analyzing the impact on HRQoL. A recent systematic review [35] concluded that previously hospitalized patients with persistent symptoms beyond 12 weeks still experienced a decline in HRQoL. Meys et al. [36] used the EQ-5D tool to determine HRQoL in non-hospitalized patients with long COVID, and obtained similar conclusions; however, they only analyzed the sample three months after diagnosis. Our study had a longer follow up, with a mean time of evolution of ten months, which may shed light on some hypotheses regarding long-term improvement in this population.

Regarding occupational balance, measured with the OBQ, this was significant for all HRQoL dimensions. Thus, those affected by long COVID showed a decline and imbalance in their occupations, which had a direct impact on their HRQoL. The relationship between occupational balance and health has been analyzed in studies with different populations and has been shown to be associated with quality of life [37]. In line with our results, recent studies have explored the relationship between occupational balance and HRQoL in the context of the pandemic situation due to COVID-19 [11]. Authors such as Messeguer de Pedro et al. [38] found low levels of occupational balance associated with a significant reduction in self-perceived health. However, we are not aware of research that has analyzed this relationship with patients with long COVID-19, even though it may be essential to train people to improve their abilities related to balancing occupational performance in order to produce a positive impact on their health and wellbeing [39]. Although we lack available evidence on the design of rehabilitation programs in this population, authors such as Belhan et al. [40] or Ganesan et al. [41] performed this intervention approach in their rehabilitation program and found improvements in HRQOL in individuals affected by the COVID-19 pandemic.

### Practical Implications and Future Lines of Investigation

Our data may be of interest for the design of appropriate and individualized intervention programs for patients with mild long COVID. In the assessment of patients with persistent COVID it is very important to consider aspects such as occupational balance, and it is necessary to design programs aimed at improving occupational balance and autonomy, which will consequently improve the quality of life of those affected.

In the future, longitudinal studies should be conducted to provide data on long-term evolution and follow-up considering other variables such as the long-term effects of vaccines in patients with long COVID who suffered a mild infection during the acute phase of illness.

## 5. Strengths and Limitations

This study aimed to fill the gap in research regarding occupational balance and its relationship with long COVID and sociodemographic variables. The reported results provide new evidence on occupational balance, a rarely studied variable in this context, and its relationship with quality of life and sociodemographic variables. This relationship has practical implications, the practitioners that attend people diagnosed with long COVID must include occupational interventions aimed to reversing the occupational imbalance, and future research should assess the effects of occupational interventions on quality of life and occupational balance.

The main strength of this research was the participant profile: people who were not vaccinated and did not require hospitalization. In the current literature, different variables related to long-term COVID have been studied in hospitalized patients who have received vaccination; however, few studies have addressed this health condition in people who have not required hospitalization or been vaccinated. In addition, the sample has been collected from different regions of Spain, and therefore, the data is more representative of the national territory. Although, the sample size was estimated to provide reliable data, the results from this study should be further confirmed in future studies with a larger sample size.

This study has several limitations which warrant consideration. Firstly, the sample size and cross-sectional design may limit the generalizability of results. Secondly, the interviews reported information from patients at two different points in time and should therefore be considered with caution to avoid possible measurement or recall bias. In spite of this, the guidelines for this type of observational study [20] were considered to minimize bias as much as possible and the interviews were scheduled soon after their previous situation. In addition, this was a commonly used methodology during pandemic periods [42,43] and at the time of the evaluation movement restrictions were still in place at a national level. Nonetheless, despite these limitations, the present study has enabled us to perform an analysis of HRQOL, as well as to make the first description of the impact of sociodemographic variables, age, sex, time of evolution, and occupational balance in patients with long COVID.

## 6. Conclusions

The results of this study indicate that people with long COVID present a low HRQOL and occupational imbalance. Furthermore, we have found that female sex, a longer time since infection, and occupational imbalance are influential variables related to a worse HRQOL.

## Figures and Tables

**Table 1:** Descriptive sociodemographic and clinical variables.

Sociodemographic Variables	*n*
Sex (*n* (%))	
Women	95 (77.9)
Men	27 (22.1)
Age (range)	30-50
Age (mean (SD))	43.5 (5.8)
Time since infection (min.–max. months (SD))	4–16 (3.33)

SD: standard deviation.

**Table 2:** Descriptive statistics for the scores on the SF-36 subscales.

	Mean (SD)
Physical Functioning (PF)	27.50 (20.40)
Role of Physical Limitations (RP)	5.12 (16.99)
General Health (GH)	29.51 (16.23)
Bodily Pain (BP)	36.52 (22.04)
Physical Component Summary (Pcs)	24.66(4.45)
Vitality (V)	22.25 (20.71)
Mental Health (Mh)	59.30 (14.94)
Social Functioning (SF)	39.45 (17.53)
Role of Emotional Limitations (RE)	62.81 (46.98)
Mental Component Summary (MCS)	45.95 (8.65)
Health Transition	7.17 (11.35)

Notes. SD: standard deviation.

**Table 3:** Means, standard deviations and correlations between scales (raw scores).

	1	2	3	4	5	6	7	8	9	10
1. Occupational Balance Questionnnaire (OBQ)	1									
2. Physical Functioning	0.61 *	1								
3. Role of Physical Limitations	−0.10	0.07	1							
4. Role of Emotional Limitations	0.29 *	0.35 *	0.19	1						
5. Vitality	0.42 *	0.60 *	0.14	0.06	1					
6. Mental Health	0.28 *	0.39 *	−0.15	0.64 *	−0.05	1				
7. Social functioning	0.39 *	0.50 *	0.03	0.38 *	0.19 *	0.47 *	1			
8. Bodily Pain	0.27 *	0.42 *	0.03	0.40 *	0.18 *	0.32 *	0.46 *	1		
9. General Health	0.53 *	0.58 *	−0.08	0.24 *	0.44 *	0.34 *	0.47 *	0.43 *	1	
10. Health Transition	0.49 *	0.46 *	0.13	0.27 *	0.38 *	0.12	0.54 *	0.33 *	0.40 *	1

Notes: * *p* < 0.05.

**Table 4:** Effect of demographic, clinical variables and OBQ on the dimensions related to physical aspects of the SF-36 scale.

	Physical Functioning			Role of Physical Health			General Health			Bodily Pain		
	B (SE)	*t*	*p*-Value	B (SE)	*t*	*p*-Value	B (SE)	*t*	*p*-Value	B (SE)	*t*	*p*-Value
Sex (Female vs. Male)	−1.03 (0.44)	−2.08	**0.040**	−0.07 (0.15)	0.45	0.656	0.44 (0.63)	0.69	0.492	−0.87 (0.41)	−2.12	0.036
Age	−0.01 (0.05)	−0.19	0.851	−0.01 (0.01)	0.65	0.518	0.02 (0.05)	0.52	0.601	0.00 (0.04)	−0.03	0.978
Time since infection	0.05 (0.02)	2.09	**0.039**	−0.02 (0.02)	1.06	0.291	0.04 (0.02)	2.11	0.037	−0.06 (0.06)	−1.05	0.296
OBQ	0.22 (0.03)	8.08	**<0.001**	−0.01 (0.01)	1.20	0.231	0.16 (0.02)	6.69	<0.001	0.05 (0.02)	3.00	0.003
R^2^ (%)	36.5			−0.8			26			7.2		
Model	F (4; 117) = 18.39; *p* < 0.001			F (4; 117) = 0.76; *p* = 0.551			F (4; 117) = 11.60; *p* < 0.001			F (4; 117) = 3.36; *p* = 0.012		

B: unstandardized coefficient. SE: standard error.

**Table 5:** Effect of demographic and clinical variables and the OBQ on dimensions related to mental health, according to the SF-36 scale.

	Vitality			Role of Emotional Limitations			Mental Health			Social Functioning		
	B (SE)	*t*	*p*-Value	B (SE)	*t*	*p*-Value	B (SE)	*t*	*p*-Value	B (SE)	*t*	*p*-Value
Sex (Female vs. Male)	−1.02 (0.86)	−1.18	0.239	−0.08 (0.31)	−0.27	0.789	0.42 (0.81)	0.52	0.604	−0.23 (0.29)	−0.79	0.432
Age	−0.03 (0.06)	−0.51	0.611	0.02 (0.02)	0.91	0.365	−0.06 (0.06)	−0.98	0.33	0.03 (0.02)	1.38	0.169
Time since infection	−0.03 (0.01)	−2.14	0.034	0.02 (0.04)	0.50	0.616	0.16 (0.10)	1.56	0.121	−0.02 (0.04)	−0.60	0.552
OBQ	0.15 (0.03)	4.75	<0.001	0.04 (0.01)	3.39	0.001	0.09 (0.03)	2.89	0.005	0.05 (0.01)	4.81	<0.001
R^2^ (%)	16			8.4			9.2			14.11		
Model	F (4; 117) = 6.78; *p* < 0.001			F (4; 116) = 3.07; *p* = 0.019			F (4; 117) = 3.35; *p* = 0.012			F (4;1 17) = 5.98; *p* < 0.001		

B: unstandardized coefficient. SE: standard error.

**Table 6:** Effect of demographic and clinical variables and OBQ on the health transition of the SF-36 scale.

	Health Transition		
	B (SE)	*t*	*p*-Value
Sex (Female vs. Male)	−5.17 (2.16)	−2.40	0.018
Age	0.20 (0.16)	1.26	0.211
Time since infection	−0.86 (0.27)	−3.13	0.002
OBQ	0.52 (0.08)	6.56	<0.001
R^2^ (%)	29.5		
Model	F (4; 117) = 13.66; *p* < 0.001		

B: unstandardized coefficient. SE: standard error.

## Data Availability

All data are available upon request from the corresponding author.
